# Metformin: Ongoing Journey with Superdrug Revolution

**DOI:** 10.15171/apb.2019.001

**Published:** 2019-02-21

**Authors:** Amr Ahmed EL-Arabey, Mohnad Abdalla, Wafa Ali Eltayb

**Affiliations:** ^1^Pharmacology and Toxicology Department, Faculty of Pharmacy, Al-Azhar University, Egypt.; ^2^Qingdao Institute of Bioenergy and Bioprocess Technology, Qingdao Shi, Shandong Sheng 266000, People’s Republic of China.; ^3^Faculty of Science and Technology, Shendi University, Shendi, Nher Anile, Sudan.

## Dear Editor,

**Figure 1:**
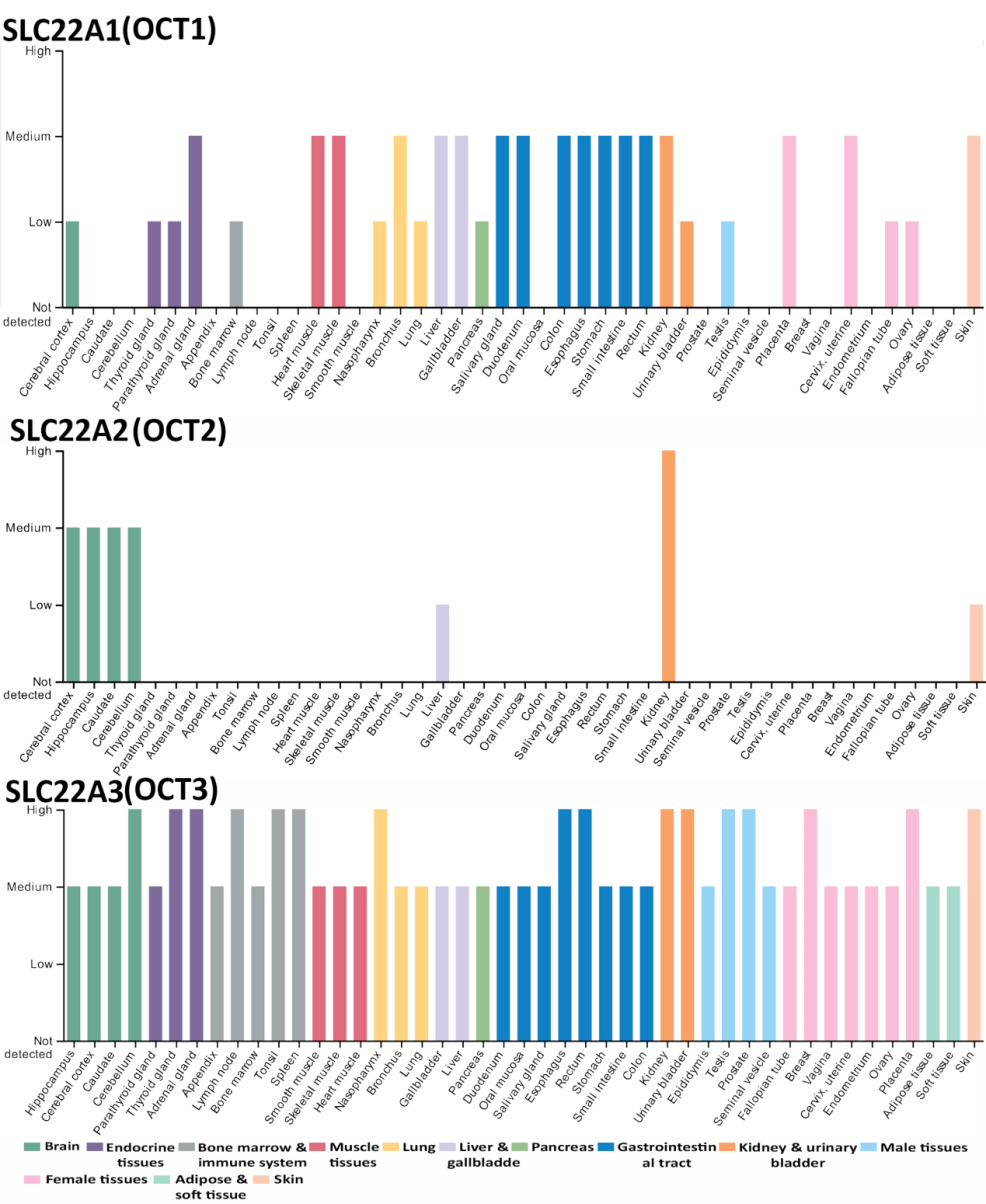
The OCT1, OCT2 and OCT3 expressions in human organs (http://www.proteinatlas.org).

Metformin a synthetic derivative of guanidine which isolated from the extracts of *Galega officinalis* in 1922 and introduced as a medication in France, England, Canada and the United States in (1957, 1957, 1972 and 1995, respectively). Metformin is a safe, essential, inexpensive medication with a story of more than 60 years. Indeed, metformin represents a worldwide superstar in therapy of patients with type 2 diabetes since its discovery.^1^ In last decade, several studies highlighted the beneficial therapeutic effects of metformin on other clinical domains such as cancer, aging, neurological diseases, cardio-vascular diseases, metabolic syndrome, Obesity and polycystic ovary syndrome. Moreover, the study conducted from 2009 to 2013 showed the off-label use of metformin among adolescents in United State approximately 6.5% diagnosed with obesity. However, further work on metformin actions for obesity could change its status to on label use by the FDA.^2^ Recent emerging evidence indicates novel actions of metformin in treatment of autoimmune disease through targeting signal transducer and activator of transcription 3 (STAT3) which responsible for macrophage polarization and reduced macrophage cytokines synthesis via inhibition of activating transcription factor 3 (ATF-3).^3^ Furthermore, the pioneer work by Piskovatska et al showed the therapeutic efficiency of metformin as a geroprotector through activation of autophagy and AMP-activated protein kinase (AMPK). In this respect, metformin act as a powerful candidate for hormesis inducing factor with pro-longevity features and health span enhancing.^3,4^ Clinically, metformin undergoing clinical trial for longevity phase 4 (NCT02432287) (clinicaltrial.gov). Moreover, metformin can be used as cytoprotective agent via reduction of apoptosis and oxidative stress.^5^ Hence, metformin act as super-drug against multiple diseases in numerous organs. Every cell has a membrane that separates the cell from the external environment, for this reason, handling of drugs mediated by a variety of different transport proteins. In this regards, transporters influences human physiology and pathophysiology and are critical key players of therapeutic response for drugs.^6^ Furthermore, a number of important human drug transporters have been noted that are distributed at the apical or basal part of the epithelial cells in several tissues to orchestrate absorption, distribution and metabolism as well as drugs excretion. Importantly, membrane transporters play a considerable role in metformin absorption, distribution, and renal excretion. Multiple organic cation transporters (OCT1, OCT2 and OCT3) are determinants of the pharmacokinetics of metformin, and many of them are important in its pharmacological action, as mediators of metformin entry into target tissues.^7,8^ The OCT1, OCT2 and OCT3 are distributed in several human organs (Figure 1) . Here we would like to shed light on metformin revolution in the current decade based on the completed and ongoing register clinical trials of metformin at (clinicaltrial.gov) (Table 1). Our analysis concerns the total clinical study of target organ of metformin according to the distribution of OCT1, OCT2 and OCT3 in human tissues. Interestingly, there are 541 completed and ongoing clinical trials of metformin on 26 target organs. However, there are no clinical trials of metformin on 11 potential target organs which can be reached by metformin such as appendix, tonsil, spleen, nasopharynx, bronchus, oral mucosa, salivary gland, small intestine, placenta, testis and soft tissue. Diverse scenarios have been manifested to illustrate the pharmacological mechanisms of metformin on different diseases for instance its metabolic effect, growth inhibition, partial inhibition of respiratory chain complex I, cell death, liver kinase B1 (LKB1)-dependent activation of AMPK, downstream of mammalian target of rapamycin (mTOR), autophagy effect, immune and hypothalamic effects.^7^ Consequently, research in needing to explore the therapeutic beneficial mechanisms of metformin in the uncovered promising target organs to draw the real picture for the ongoing revolution journey of metformin as super star drug.

**Table 1:** Recorded clinical trials for metformin

**Organ**	**Total of study**	**Start time**	**Conditions**	**Study has results**	**Transporter**
Brain	34	August 2001	Brain tumor treated with cranial, cranial-spinal radiation, brain neoplasms, brain cancer, primary brain tumors, mild cognitive impairment, amnestic mild cognitive impairment, movement disorders, glioblastoma multiforme, Li-Fraumeni syndrome, Alzheimer's disease, vascular dementia, dementia, memory impairment, schizophrenia, depression, migraine.	7	OCT1 (cerebral cortex), OCT2 (cerebral cortex, hippocampus, Caudate, Cerebellum) and OCT3 (cerebral cortex, hippocampus, Caudate, Cerebellum)
Thyroid gland and parathyroid gland	3	April 2010	Malignant neoplasm of thyroid stage I & II, thyroid hormone metabolism in euthyroid patients, mitigating side effects of radioactive iodine treatment in patients with differentiated thyroid cancer.	-	OCT1 (thyroid gland, parathyroid gland), and OCT3 (thyroid gland, parathyroid gland)
Adrenal gland	1	December 2016	Hyperandrogenemia	-	OCT1 and OCT3
Appendix	-	-	-	-	OCT3
Tonsil	-	-	-	-	OCT3
Bone marrow	1	July 2013	Adult acute megakaryoblastic leukemia (M7), adult acute minimally differentiated myeloid leukemia (M0), adult acute monoblastic leukemia (M5a), adult acute monocytic leukemia (M5b), adult acute myeloblastic leukemia with maturation (M2), adult acute myeloblastic leukemia without maturation (M1), adult acute myeloid leukemia with 11q23 (MLL) abnormalities, adult acute myeloid leukemia with del(5q), adult acute myeloid leukemia with inv(16)(p13;q22), adult acute myeloid leukemia with t(16;16)(p13;q22), adult acute myeloid leukemia with t(8;21)(q22;q22), adult acute myelomonocytic leukemia (M4), adult erythroleukemia (M6a), adult pure erythroid leukemia (M6b) blastic phase chronic myelogenous leukemia, recurrent adult acute myeloid leukemia, untreated adult acute myeloid leukemia.	-	OCT1 and OCT3
Lymph node	2	March 2008	Lymphoma, metastatic melanoma (stage IIIC non-resectable or no surgically curable or stage IV).	-	OCT3
Spleen	-	-	-	-	OCT3
Heart Muscle	12	May 2000	Heart Failure, left ventricular hypertrophy, coronary artery disease, ischemic heart disease, peripheral arterial disease, intermittent claudication, fibromyalgia mitochondrial diseases, movement disorders.	1	OCT1, OCT3
Skeletal Muscle	11	May 2000	Physical inactivity and insulin resistance in skeletal muscle, studies in skeletal muscle in case of insulin sensitivity on reproductive function in polycystic ovary syndrome, muscle energetics, and vascular function in older adults with peripheral artery disease, muscle mitochondrial dysfunction in diabetes, defective atypical protein kinase C activation in diabetes and metabolic syndrome.	1	OCT1, OCT3
Nasopharynx	-	-	-	-	OCT1, OCT3
Bronchus	-	-	-	-	OCT1, OCT3
Lung	14	March 2008	Lung Cancer, lung neoplasms, idiopathic pulmonary arterial hypertension, heritable pulmonary arterial hypertension, scleroderma associated pulmonary arterial hypertension, NSCLC, EGFR gene amplification, advanced cancer stage IIIB NSCLC, stage IV NSCLC, adenosquamous lung carcinoma, bronchioloalveolar carcinoma, large cell lung carcinoma, lung adenocarcinoma non-small cell lung carcinoma, recurrent non-small cell lung carcinoma, squamous cell lung carcinoma, stage IIIA & IIIB Non-small cell lung cancer, chronic obstructive pulmonary disease.	1	OCT1, OCT3
Liver	92	October 2000	Fatty liver, liver diseases, non-alcoholic fatty liver disease, liver cirrhosis, hepatic encephalopathy, hepatocellular carcinoma, hepatitis c, chronic cirrhosis, NASH related cirrhosis, hepatitis, steatohepatitis, abnormal liver function tests, intrahepatic cholestases, hepatic steatosis, acute liver injury.	9	OCT1, OCT2, OCT3
Gallbladder	2	July 2012	Gallbladder emptying on postprandial glucagon-like peptide-1(GLP-1) secretion in type 2 diabetes.	-	OCT1, OCT3
Pancreas	64	May 2000	Stage IA, IB, IIA and IIB pancreatic adenocarcinoma, resectable pancreatic ductal adenocarcinoma, pancreatic cancer, acinar cell adenocarcinoma of the pancreas, recurrent pancreatic cancer, stage I-III pancreatic cancer, locally advanced pancreatic cancer, metastatic pancreatic cancer, unresectable pancreatic carcinoma, partial pancreatectomy resulting from benign pancreatic neoplasm.	11	OCT1, OCT3
Duodenum	3	October 2012	Metabolic effects of duodenal jejunal bypass plus sleeve gastrectomy in T2DM, familial adenomatous polyposis, morbidly obese adolescent with a duodena-jejunal liner.	0	OCT1, OCT3
Oral mucosa	-	-	-	-	OCT3
Colon and rectum	15	March 2011	Advanced colorectal cancer, recurrence of non-DM stage III colorectal cancer, refractory colorectal cancer, colorectal neoplasms, adenocarcinoma, metastatic colorectal cancer, adenomatous polyp, colorectal cancer, familial adenomatous polyposis, colon cancer, tertiary prevention in colon cancer.	2	OCT1, OCT3
Salivary gland	-	-	-	-	OCT1, OCT3
Esophagus	2	May 2010	Short segment Barrett's esophagus, long segment Barrett's esophagus, esophageal cancer.	1	OCT1, OCT3
Stomach	1	September 2009	Gastric bypass	-	OCT1, OCT3
Small intestine	-	-	-	-	OCT1, OCT3
Kidney	50	September 1999	Polycystic kidney, autosomal dominant, chronic kidney disease, renal insufficiency, chronic renal insufficiency, renal and vascular changes in type 2 diabetes, genetic basis for variation in the metformin renal elimination, reduced renal function, systemic and renal endothelial function, acute kidney injury, kidney cancer, kidney transplantation, chronic kidney disease, diabetic nephropathy, congenital nephrogenic diabetes insipidus.	-	OCT1, OCT2, OCT3
Urinary bladder	1	October 2015	Bladder cancer	-	
Prostate	26	June 2009	Prostate cancer, prostate cancer recurrent, prostatic neoplasms, metastatic prostate cancer, adenocarcinoma of the prostate, stage I, IIA, IIB & III prostate cancer.	2	OCT3
Testis	-	-	-	-	OCT3
Placenta	-	-	-	-	OCT3
Breast	38	October 2008	Breast cancer, metastatic breast cancer, hormone receptor positive malignant neoplasm of breast, stage 0 breast carcinoma, breast neoplasms, stage I breast carcinoma, stage II breast carcinoma, stage III breast carcinoma,HER-2 positive breast cancer, locally advanced malignant neoplasm, human epidermal growth factor 2 negative carcinoma of breast, atypical ductal breast hyperplasia, BRCA1 & BRCA2 mutation carrier, ductal breast carcinoma in situ, lobular breast carcinoma in situ.	1	OCT3
Vagina	1	January 2013	Vaginal administration of metformin in Polycystic ovary syndrome patients.	-	OCT3
Cervix, Uterine	1	May 2015	Uterine cervical neoplasms, squamous cell carcinoma, adenocarcinoma, carcinoma, aden-squamous.	-	OCT1, OCT3
Endometrium	8	September 2010	Adenocarcinoma of the endometrium, endometrial cancer, complex endometrial hyperplasia with atypia, grade 1 endometrial endometrioid adenocarcinoma.	-	OCT3
Fallopian tube	3	October 2011	Fallopian tube cancer, stage IIIA fallopian tube cancer, stage IIIB, IIIC & IV fallopian tube cancer, recurrent fallopian tube cancer.	-	OCT1, OCT3
Ovary	124	January 2000	Polycystic ovary syndrome, anovulation, infertility, endothelial dysfunction, reproductive endocrinology, hyperandrogenism, in vitro fertilization, recurrent miscarriage.	4	OCT1, OCT3
Adipose tissue	26	May 2000	Chronic inflammatory activation in fat tissue, obesity, metabolic syndrome, childhood obesity, lipodystrophy, liver fat content and visceral fat mass in overweight and obese type 2 diabetes patients, non-alcoholic fatty liver disease, pre-diabetes mellitus and obesity, HIV lipodystrophy.	7	OCT3
Soft tissue	-	-	-	-	OCT3
Skin	6	February 2009	Aging, psoriasis, acanthosis nigricans, psoriatic arthritis, chronic foot ulcers.	1	OCT1, OCT2, OCT3

Source: https://clinicaltrials.gov.

## Ethical Issues

Not applicable.

## Conflict of Interest

There is no conflict of interest to declare.
